# A comprehensive comparative study of generative adversarial network architectures for synthetic computed tomography generation in the abdomen

**DOI:** 10.1002/mp.18038

**Published:** 2025-08-13

**Authors:** Mariia Lapaeva, Agustina La Greca Saint‐Esteven, Philipp Wallimann, Nicolaus Andratschke, Matthias Guckenberger, Manuel Günther, Stephanie Tanadini‐Lang, Riccardo Dal Bello

**Affiliations:** ^1^ Department of Radiation Oncology University Hospital Zurich and University of Zurich Zurich Switzerland; ^2^ Artificial Intelligence and Machine Learning Group Department of Informatics University of Zurich Zurich Switzerland; ^3^ Computer Vision Laboratory ETH Zurich Zurich Switzerland

**Keywords:** deep learning, generative adversarial networks, medical image analysis, MR‐only radiotherapy, synthetic CT

## Abstract

**Background:**

Magnetic Resonance (MR)‐based synthetic Computed Tomography (sCT) generation is an emerging promising technique, required for the transition from conventional planning workflows to MR‐only radiotherapy planning. This shift aims to replace CT acquisition with a sCT improving both cost efficiency and burden to the patient. Generative Adversarial Networks (GANs) have shown some of the best performance in this area.

**Purpose:**

This study aims to identify optimal approaches to improve the quality and clinical applicability of MR‐based sCT generation for treatment planning by performing an extensive comparison of GAN architectures and parameters thereof. It focuses on the abdominal region, which still lacks certified medical products for sCT generation.

**Methods:**

In order to improve the current state of deep learning technologies, we generated sCTs based on abdominal MR images of 154 cancer patients using GANs, varying the following parameters: (1) generator architectures (U‐Net, ResNet); (2) GAN architectures trained in paired (Pix2Pix) and unpaired fashion (CycleGAN and CUT); (3) number of input‐output channels (2D, 2.5D); (4) training set size. The quality of sCT generation was assessed by using both image similarity and dosimetric metrics; correlation between the two was evaluated. The dosimetric accuracy was evaluated through an automated process that compared the dose distributions of photon treatment plans calculated on sCT and CT images, using Dose‐Volume Histogram (DVH) parameters for tumor and organs at risk.

**Results:**

The Pix2Pix model, trained in paired fashion with 2.5D input‐output channels and a ResNet generator emerged as the best‐performing model, achieving a mean absolute error (MAE, mean) of 63.21 HU, a planning target volume Dmean difference of ‐0.09%, and no outliers above 2% for other DVH parameters. This configuration addressed prior challenges of Pix2Pix with bone and rigid organ boundary generation, delivering robust results even for cases with significant air pockets. The 2D input‐output channel configuration showed beneficial for GANs trained in unpaired fashion, achieving a mean MAE of 66.97 HU for CycleGAN and 69.49 HU for CUT. Both delivered clinically applicable results, with mean DVH discrepancies below 0.8%. Expanding the training set size was essential for minimizing outliers in dosimetric parameters. High correlation was observed between the image similarity metrics—MAE, MAE bones, structural similarity index measure—and target DVH parameters, with Pearson coefficients ranging from 0.77 to 0.9. However, within the clinically relevant range of DVH deviations (± 2%), stochastic variations obscured linear trends.

**Conclusions:**

The study provided a new benchmark for the abdominal sCT generation task, showing its clinical applicability for treatment planning and further advancing the state‐of‐the‐art. This study also confirmed that image similarity metrics alone can not reliably predict small dosimetric deviations within a clinical threshold; but contributed by identifying specific metrics that correlate with DVH discrepancies above ± 5%, offering valuable tools for training, evaluation, and standardization of reporting across studies.

## 1 INTRODUCTION

Modern treatment planning involves multimodal imaging, using Magnetic Resonance (MR) along with Computed Tomography (CT) images to gather essential information about patient anatomy and tissue properties. While MR images are superior for the tumor and organs delineation, CT images enable direct electron density map estimation from Hounsfield Units (HU), required to calculate dose distribution. However, CT imaging mandates a dedicated patient appointment, necessitates image‐to‐image registration with other modalities, introduces additional radiation exposure to patients and additional cost. Synthetic CT (sCT) generation emerged as a potential solution, promising advancements in radiotherapy planning and facilitating the transition towards MR‐only radiotherapy.^1^ This shift envisions the replacement of CT acquisition with an accurate sCT generation method, enhancing both cost‐effectiveness and patient care.^2^

Previous methods for sCT generation include bulk density^3^, ^4^ atlas‐based^5^, ^6^ sequence‐based^7^, ^8^ and deformable registration approaches.^9^ However, these approaches often encounter challenges in accurately reproducing anatomical variations. They can also be sensitive to image registration accuracy, segmentation errors, and assumptions made during the mapping or conversion processes, potentially resulting in errors of up to 2–5 mm for various treatment sites.^10^ Moreover, these methods frequently require manual interventions and the expertise of expensive specialists to fulfill their intended objectives.

Deep learning methods have emerged as the state‐of‐the‐art approach for sCT generation^11^ with Generative Adversarial Networks (GANs) being among the most popular and reliable architectures.^12^ They consist of two networks, a generator and a discriminator, which compete against each other to produce realistic synthetic images. In cancer treatment, where time and precision are critical, current practices often involve patients waiting in treatment positions for plan adjustments. GANs address this by generating sCT images in just 9 s for a 3D volume of 160 slices, compared to 10 min with atlas‐based methods.^13^

Currently, the most widely used GAN architectures include Image‐to‐Image Conditional Generative Adversarial Network (Pix2Pix)^14^ and Cycle‐Consistent Generative Adversarial Network (CycleGAN).^15^ While Pix2Pix requires co‐registered image pairs, CycleGAN operates on images from each modality without the need for perfectly aligned pairs from the same patient. Due to limited paired CT‐MR data availability, the architectures that do not require perfectly aligned image pairs are of high importance. Recently introduced Contrastive Learning for Unpaired Image‐to‐Image Translation (CUT)^16^ uses a patch‐based approach for translation without requiring image alignment. Unlike Pix2Pix, which requires pixelwise correspondence, and CycleGAN, which employs cycle consistency, CUT leverages contrastive learning to maximize mutual information between corresponding patches in input and output images, enabling efficient one‐way mapping. CUT has proven successful in various translation tasks, but its application for sCT generation has, to our knowledge, only been demonstrated once in the brain,^17^ and particularly the abdomen and other areas are still unexplored,^12^ motivating ongoing research efforts.

As shown by most recent literature reviews on deep learning methods for sCT generation,^11^, ^12^, ^18^, ^19^, ^20^ the abdomen remains one of the least studied anatomical areas. While there are commercial solutions are available for sCT generation for head and neck (MRCAT, Philips, Eindhoven, The Netherlands); brain and pelvis (TheraPanacea, Paris, France); prostate and brain (syngo.via, Siemens, Erlangen, Germany); brain, pelvic, head and neck (Spectronic Medical AB, Helsingborg, Sweden); the considerable differences due to the non‐rigid organs and air‐pocket movements in abdominal cavity pose additional challenges. In addition, the proportion of studies investigating the generation of sCT based on low‐field MRI (0.3‐1T) is only 6.5%.^11^ Therefore, it requires further investigation.

In the realm of CT synthesis, researchers emphasize the influence of various image normalization techniques,^21^, ^22^, ^23^ as well as different Neural Network (NN) configurations such as architecture,^17^, ^22^, ^24^, ^25^, ^26^ number of input/output channels,^27^, ^28^, ^29^ loss function,^23^, ^27^, ^30^ and other parameters.^30^, ^31^ Despite this, there is a lack of comprehensive studies focusing on multiple technical aspects. A notable exception is the study^21^ assessing the impact of the key parameters on the sCT quality of brain images. Moreover, researchers underline that there is no unified approach to evaluating the quality of generated sCT.^12^, ^21^ The literature lacks studies that investigate the relationship between image‐related quality metrics of sCT with metrics that are relevant for the evaluation of radiotherapy plans in clinical practice, such as dose‐volume histogram (DVH) parameters.^32^ The absence of consensus on optimal network architectures to capture MR‐to‐CT transformation and the misalignment of assessment techniques continue to drive our research efforts.

Therefore, the goal of our research is to perform an extensive comparative study of GAN architectures for synthetic CT generation specifically tailored to the abdominal area. Our aim is to identify optimal approaches to enhance the quality and clinical applicability of MR‐based sCT image generation by investigating diverse technical aspects.

## 2 MATERIALS AND METHODS

### 2.1 Dataset

Imaging data of 154 patients with primary tumor or metastasis in abdomen treated at the University Hospital Zurich between August 2020 and May 2022 were retrospectively analyzed in this study. The exclusion criteria were age below 18 years and the presence of implants or contrast agents. In order to improve model generalization, no further exclusion criteria has been applied. This resulted in 197 MR‐CT co‐registered volumes as some patients had multiple courses of treatments. Subsequently, the dataset was split into patient groups stratified by cancer treatment side in a random fashion, with 80% allocated to the training set and 20% to the testing set, resulting in 160 and 37 image pairs, respectively (Table S1). Patients who underwent multiple treatments were only included in the training set. All data was anonymized. All institutional guidelines were followed. Informed consent was obtained from all patients. The study was approved by the cantonal ethics committee Zurich (BASEC‐Nr. 2018‐01794).

### 2.2 Image data acquisition and preprocessing

The MR images were acquired using True Fast Imaging with Steady‐state Precession (TrueFISP) pulse sequence at the 0.35 T MRI scanner of the MRIdian system (ViewRay, Ohio, USA). The planar resolution ranged from 1.49 to 1.63 mm depending on the Field Of View (FOV) and the axial resolution was 3 mm. Axial coverage spanned up to 24 cm (80 slices), with images acquired during Expiratory Breath‐Hold (EBH). The CT images were acquired using a Somatom Definition AS (Siemens, Erlangen, Germany) scanner operated at 120 kVp, following the same immobilization and EBH setup.

The CT acquisition was performed typically within 60 min after MR. The planar resolution ranged from 0.98 to 1.63 mm depending on the FOV and the axial resolution ranged from 1 to 2 mm. The CT images were imported to the MRIdian TPS and registered to MR with the help of the embedded image registration software with deformable registration, using default setting.^33^, ^34^ These CT images are further referred to as deformed CT (dCT). The registrations were then reviewed and approved by an experienced radiation oncologist who assessed the positioning and deformation of Organs At Risk (OAR) and targets. In addition, a medical physicist reviewed the registrations to ensure that the FOV was suitable and to assess the positioning and deformation of hyper‐ and hypo‐dense regions, which is critical for accurate dose calculation.

In a subsequent preprocessing step, both the MR and dCT volumes were resampled using spline interpolation to match the modal resolution of 3 × 1.63 × 1.63 mm^3^. Due to the signal loss for the slices at the edge of FOV, only 20 axial slices were taken from each volume around the tumor center in both directions after careful examination of the tumor sizes. This allowed coverage of the Planning Target Volumes (PTVs) and the 2 cm margin while excluding corrupted slices that are not relevant for dose calculation. As a result, all volumes were resized to 40 × 256 × 256 voxels. To expand the intensity range in the Region Of Interest (ROI), similarly to,^21^, ^35^ intensity values of dCT images were clipped to the HU range from air to cortical bone (1200) and, then, rescaled linearly to [0,1]. For MR images, no adjustments to intensity values beyond linear scaling to the range [0,1] were applied, referred to as “No normalization”.

### 2.3 Neural network architecture and training

In order estimate the influence of architecture on the quality of generated sCT, Pix2Pix^14^ architecture was trained in paired fashion (on co‐registered image pairs), while CycleGAN^15^ and CUT^16^ were trained in unpaired fashion, requiring only images from each modality and similar body regions, reproducing the proposed implementations.^36^, ^37^ Depending on the experiment, a U‐Net^38^ or a ResNet^39^ generator was used together with a PatchGAN discriminator.^14^ The U‐Net generator uses skip connections that directly add encoder downsampling outputs to decoder upsampling inputs, while ResNet's residual connections bypass two consecutive convolutions layers between a few downsampling∖upsampling operations thus “flattening” the model (Figure S2).

The training of networks was performed in 2D: passing axial slices to the single‐channel input and output; or in 2.5D fashion: by passing 3 adjacent axial slices as an input and 3 generated as an output with stride one in axial direction and then applying median fusion strategy (Figure S3). The median fusion strategy was chosen due to its effectiveness according to the results of our initial research.^40^ The model training was carried out on a high performance cluster with Nvidia GeForce 1080 Ti (11 GB RAM) GPU and 50 GB of allocated memory, for 100 epochs, using the PyTorch framework.^41^ Hyperparameters used for training of NN models could be found in Figure S4.

### 2.4 Experimental objectives and key configurations

**FIGURE 1:**
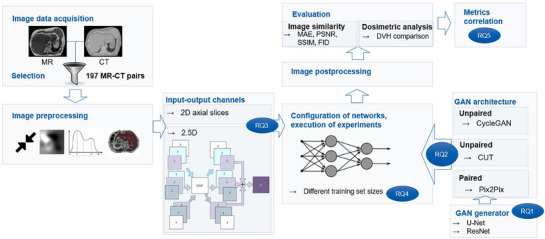
Schematic representation of the study design. The study is aimed to answer five main research questions, regarding (RQ1) the generator architectures for producing sCTs, (RQ2) GAN architectures trained in paired and unpaired fashion, (RQ3) the number of input‐output channels, (RQ4) the impact of the training set size and (RQ5) whether image similarity metrics correlate with dosimetric metrics.

To achieve the study objective of optimizing the GAN‐based sCT generation in order to improve its clinical applicability for abdominal treatment planning, the study was sub‐divided into addressing five research questions. The schematic representation of the study design can be found in Figure 1.

The first research question (RQ1) was set to analyze whether the use of ResNet as the generator in GANs could enhance the fidelity and quality of sCT images by leveraging efficient residual feature learning compared to the commonly used U‐Net generator. To investigate this, the first experiment was conducted to compare the performance of two Pix2Pix models employing PatchGAN discriminator and U‐Net or ResNet generator. In this and all subsequent experiments, models were trained in 2D fashion, with no normalization applied to MR images.

The second research question (RQ2) was to quantify the influence of NN architectures, trained in unpaired fashion (CycleGAN, CUT) as compared to architectures like Pix2Pix, which require perfectly aligned image pairs.

The third research question (RQ3) aimed to estimate the role of NN input‐output channels configuration: whether a NN trained with the help of three adjacent 2D slices could avoid 3D discontinuities in the abdomen, which is heavily affected by respiratory and physiological changes. In the third experiment, each architecture (Pix2Pix, CycleGAN, CUT) was trained using both 2D and 2.5D input‐output configurations.

The fourth research question (RQ4) explores how increasing the training set size impacts the evolution of sCT quality metrics, including the occurrence of dosimetric outliers. To address this, the fourth experiment involved training all architectures in second experiment configuration on varying amounts of training data—8, 10, 16, 32, 50, 64, 128 as well as all 160 3D image volumes used as the standard training set size for all other experiments. The test cohorts remained the same for all models.

The final research question (RQ5) targeted to determine whether there is a correlation of image similarity metrics with the dosimetric metrics, which may be potentially used for evaluating the quality of the generated sCTs. In the fifth experiment, the metrics of all models from previous experiments were compared using Pearson^42^ and Spearman^43^ correlation coefficients to assess both linear and non‐linear relationships, including metrics from experiment 4 with various training set size configurations to analyze trends in larger error areas. The interpretation of correlation coefficients follows the conventional approach described by Schober et al.^44^

### 2.5 Evaluation of synthetic CT quality

The quality of generated sCT images was evaluated with the help of both image similarity metrics between the sCT and the dCT and dosimetric metrics.

As of the image similarity metrics, which help to compare voxel values and geometry of the images, Mean Absolute Error (MAE), Mean Squared Error (MSE), Peak Signal‐to‐Noise Ratio (PSNR), Structural Similarity Index Measure (SSIM) were evaluated on the slice level within the body contour, similarly to Hsu et al.^45^ In addition, the MAE metric was calculated for the bone area (MAE bones) and the body area, excluding air pockets (MAE no air) determined by the following thresholding technique. A threshold of 250 HU was used for the MAE bones and of ‐ 400 HU for the MAE no air to exclude from evaluation potential location gaps in air pocket positions between MR and dCT. Fréchet Inception Distance (FID)^46^ was calculated as well.

To calculate the dosimetric metrics, the temporal gaps between the acquisition of dCT and MR images were compensated in a first step. Distortions in rigid organs and air pockets were addressed by bulk density override with those of soft tissue (7 HU) and air (‐1024 HU) on dCT using a mask manually contoured by the expert. No bulk density override was applied to the generated sCT images to assess the efficiency of GANs, trained in paired and unpaired fashion, in reproducing the required structure and the possibility of excluding additional time‐consuming manual steps.

Following compensation, realistic dose distribution plans were calculated with the Photon Treatment Plan module of matRad, an open‐source dose calculation and optimization toolkit.^47^ We optimized a realistic plan based on a SBRT prescription of 5 × 8 Gy at 65%. For this purpose, tissue and tumor masks contoured on the original image volumes were extracted. The fluence optimization parameters for Gross Tumor Volumes (GTVs) or Clinical Target Volumes (CTVs) included a penalized minimum DVH target with dose of 54 Gy to 95% volume. The PTVs were optimized with a MinMaxDose target ranging from 40 Gy (minimum) to 62.4 Gy (maximum) dose. As for constraints, restrictions were applied to OARs, with a penalized maximum DVH target (30 Gy to 1% of volume) for the stomach, duodenum and bowel; a penalized maximum DVH target (12 Gy to 1% of volume) for the spinal cord and a penalized mean dose target of 12 Gy for the liver. As of the beam parameters, the bixel width was set to 4 mm, the number of fractions to 5, the gantry angles passed as a vector (0 23 50 75 95 110 150 170 190 210 250 265 285 310 337), the couch angles to 0 and the dose grid size set to 4 × 4 × 4 mm^3^. The plans originally optimized on the dCT were rigidly copied and recalculated on the sCT with the same parameters.

The DVH comparison for target structures included the target coverage (PTV D95%), near‐maximum (PTV D2%), near‐minimum (PTV D98%), and mean dose (PTV Dmean and GTV/CTV Dmean).^22^ The OAR were evaluated through the near‐maximum dose (Stomach D2%, Duodenum D2%, Bowel D2%, Spine D2%) and the mean dose (Ipsilateral Kidney Dmean, Liver Dmean). These metrics and their definitions were selected in accordance with ICRU guidelines.^48^ The dosimetric points calculated on the sCT were compared to their value on the dCT. The number of outliers from the clinically relevant range of DVH deviations^49^ beyond ± 2% was estimated.

Depending on the experiment, non‐parametric statistical tests (Table S5) were performed with the repeated measures design to determine whether the differences in the distribution of DVH parameters between dCT and sCT calculated plans were significant (*p* < 0.05)^44^: asymptotic Wilcoxon‐Pratt signed‐rank test, Friedman test or Permutational Multivariate Analysis of Variance (PERMANOVA). In addition to the statistical methods, a shuffle‐split cross‐validation was performed to check the stability of the network performance for the fourth experiment due to the largest coverage of possible deviations between the models. Code pipeline, including the possibility to automate the sCT‐dCT dose difference calculations is available here: https://github.com/medical‐physics‐usz/synthetic_CT_generation.

## 3 RESULTS

### 3.1 Experiment 1: Effect of generator architecture (RQ1)

**FIGURE 2:**
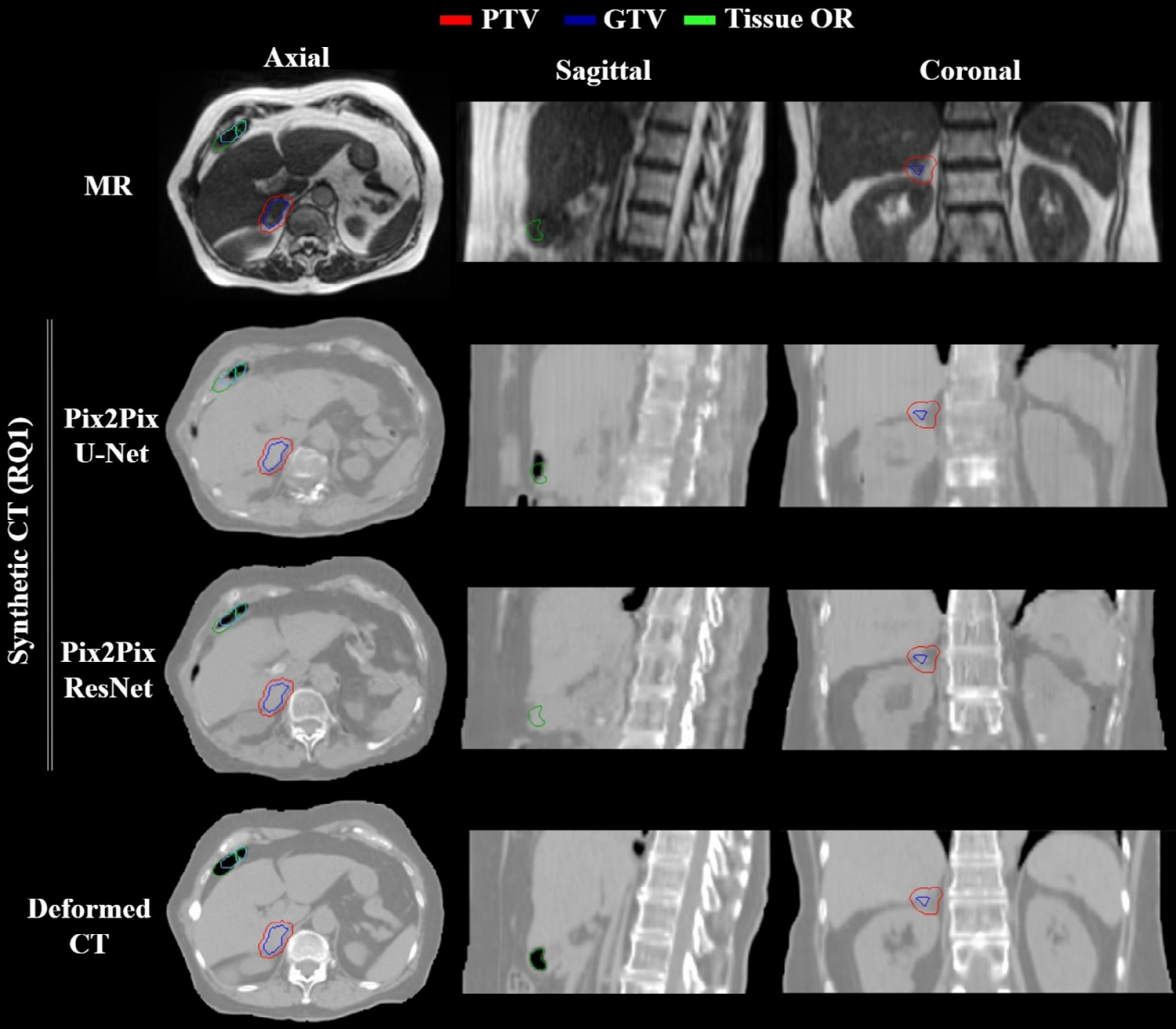
Research question 1. Example of sCT volumes generated with the help of U‐Net and ResNet generators for an adrenal gland case. The axial and sagittal slices reveal the ability of the ResNet to obtain sharper organ boundaries while outperforming U‐Net in the ability to avoid manual air and soft tissue bulk density override (Tissue OR) steps by automatically filling these structures correctly. The coronal slices show the performance in the bone region. The window width and window level of the shown CT and sCT images are 844 and ‐100 HU.

**FIGURE 3:**
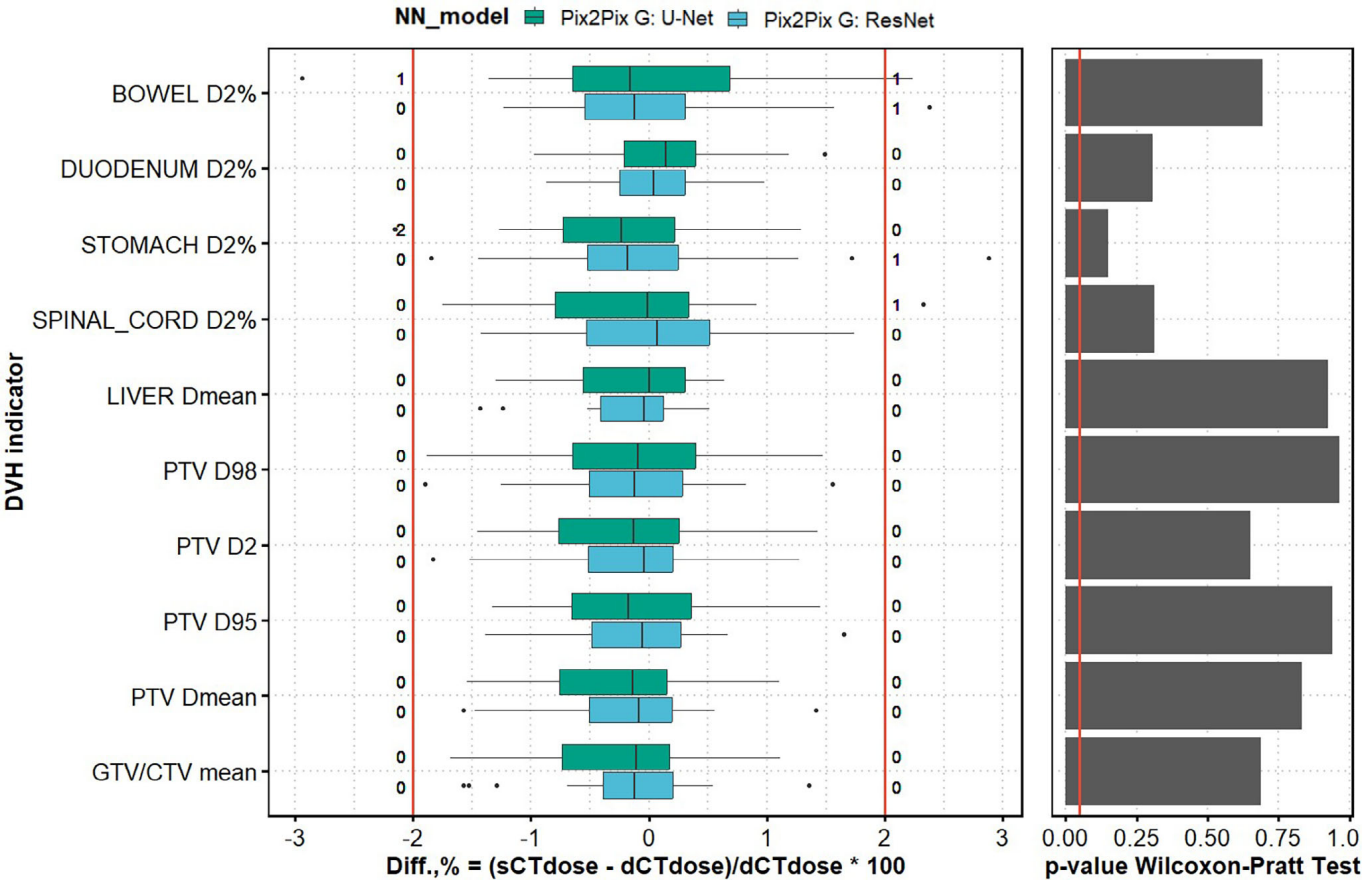
Research question 1. Differences in DVH dosimetric indicators between plans calculated on dCT and sCT, generated with Pix2Pix models, employing U‐Net and ResNet generators. The number of outliers below ‐2% and above 2% is shown next to the red lines for each DVH indicator. The right panel reports the results of the Wilcoxon‐Pratt test, with each DVH parameter evaluated independently (Table S5). The significance level *p* = 0.05 is highlighted with a vertical line and no values below are observed.

A representative case showing the sCT volumes generated using U‐Net and ResNet generators in the Pix2Pix model is shown in Figure 2. The sCT generated using the ResNet generator showed sharper boundaries and better similarity to the dCT in dense structures. Overall, both models showed comparable results, based on image similarity metrics (Table 1, see RQ1 columns). However, ResNet showed smaller error in the bone area (MAE bones) of 264.97 ± 49.53 HU, compared to 313.35 ± 52.05 for U‐Net and twice the decrease of FID. The summary of the evaluated DVH dosimetric indicators is reported in Figure 3. While the significance of statistical differences was not found (*p *> 0.05), more outliers were observed for the U‐Net model.

**TABLE 1:** Quantitative analysis of image similarity between sCT and dCT for the Pix2Pix model (trained in paired fashion) and for the CycleGAN and CUT models (trained in unpaired fashion) with no normalization applied to MR images. Units are in HU for MAE, HU^2^ for MSE, dB for PSNR. Bold values show the best performance within an architecture, while underlined metric values show the best performance across all architectures. Unless otherwise specified (G: U‐Net), all models utilize a ResNet generator.

	Pix2Pix			CycleGAN		CUT	
Metric (Mean ± SD)	2D (G: U‐Net)	2D	2.5D	2D	2.5D	2D	2.5D
RQs	RQ1,5	RQ1,2,3,4,5	RQ3,5	RQ2,3,4,5	RQ3,5	RQ2,3,4,5	RQ3,5
MAE	68.75 ± 18.26	67.58 ± 18.99	**63.21 ± 18.47**	**66.97 ± 18.81**	78.95 ± 21.44	**69.49 ± 19.40**	72.41 ± 19.05
MAE no air	49.66 ± 9.61	48.25 ± 10.33	**44.59 ± 9.91**	**46.20 ± 9.97**	52.60 ± 9.42	**48.15 ± 9.91**	50.23 ± 9.66
MAE bones	313.35 ± 52.05	264.97 ± 49.53	**251.46 ± 51.03**	**264.33 ± 52.69**	316.76 ± 50.60	**259.93 ± 50.25**	280.32 ± 53.57
MSE	2242.60 ± 495	**2212.43 ± 512**	18823 ± 10592	**2041.04 ± 536**	27299 ± 12872	**2112.66 ± 467**	22528 ± 11211
PSNR	38.84 ± 0.92	**38.90 ± 0.95**	30.07 ± 2.18	**39.27 ± 1.09**	28.30 ± 1.88	**39.09 ± 0.90**	29.15 ± 1.89
SSIM	0.983 ± 0.009	0.983 ± 0.009	**0.985 ± 0.009**	**0.983 ± 0.009**	0.976 ± 0.012	**0.982 ± 0.010**	0.981 ± 0.010
FID	51.17	**22.87**	27.40	**18.19**	52.25	**18.07**	22.80

### 3.2 Effect of NN architectures, trained in paired, and unpaired fashion (RQ2)

**FIGURE 4:**
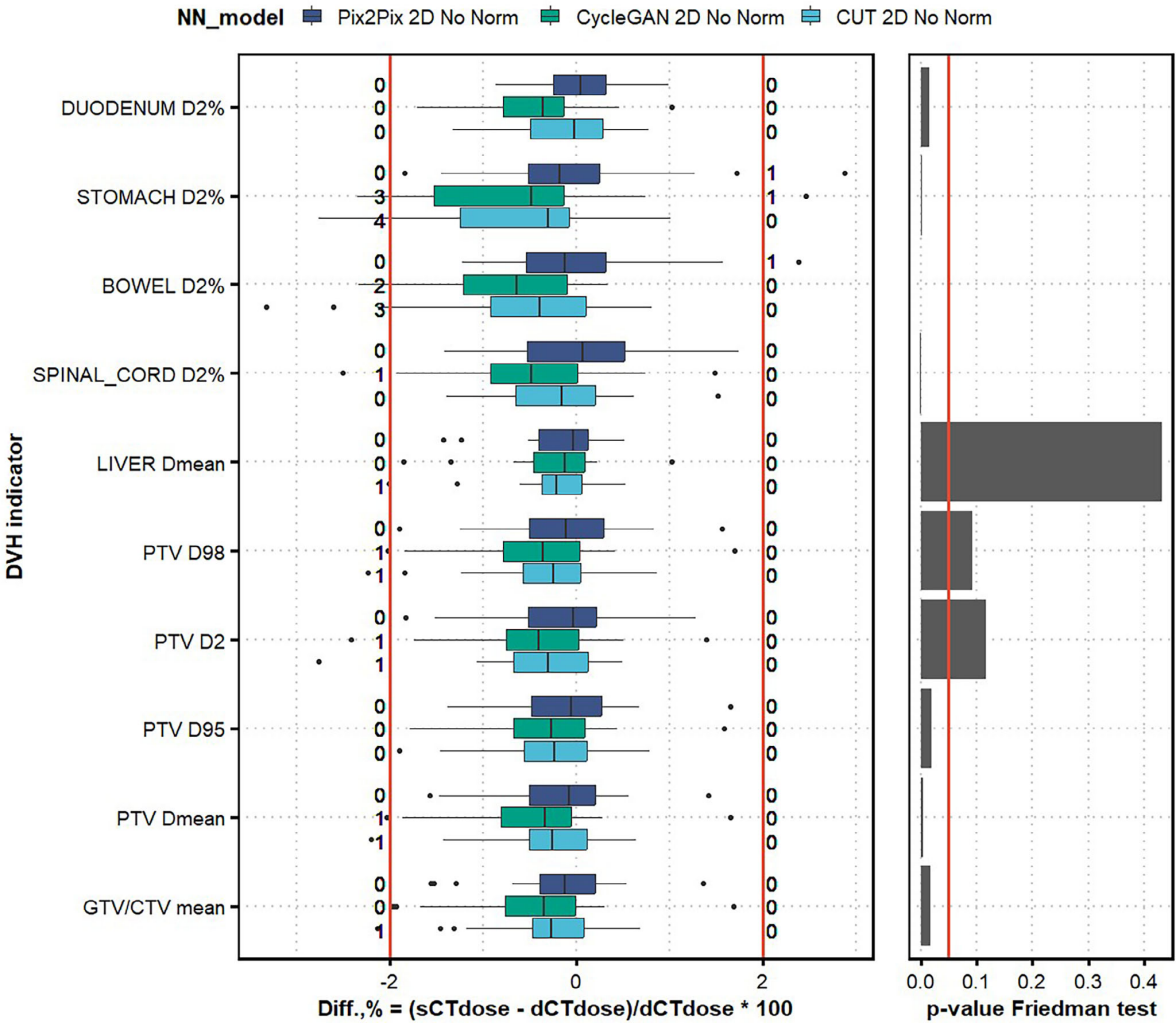
Research question 2. Differences in the dosimetric DVH indicators between plans calculated on dCT and sCT, generated with the Pix2Pix model, trained in paired fashion, and unpairedly‐trained CycleGAN and CUT. The number of outliers below ‐2% and above 2% is shown next to the red lines for each DVH indicator. The right panel reports the results of the Friedman test, with each DVH parameter evaluated independently (Table S5). The significance level *p* = 0.05 is highlighted with a vertical line.

**FIGURE 5:**
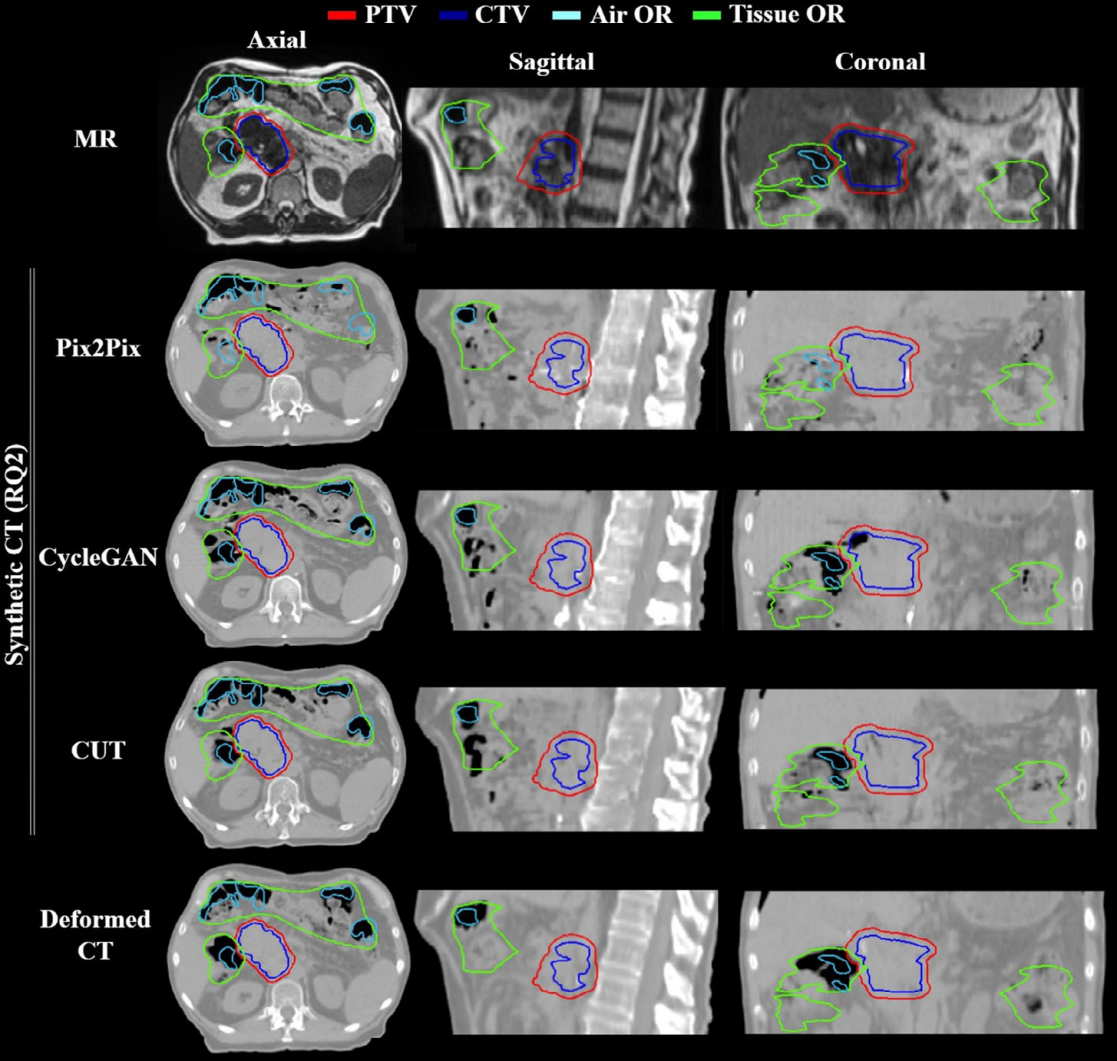
Research question 2. Example of sCT volumes generated with the help of architectures trained in paired (Pix2Pix) and unpaired fashion (CycleGAN, CUT) for a pancreas case, *having an extreme case of air volume*. Air OR and Tissue OR contours define regions for air and soft tissue density overrides (OR), ensuring correct placement on deformed CT relative to MR for treatment planning. The coronal slices show the superior results of the unpaired trained architectures in rib formation. While Pix2Pix is less accurate in generating the air pocket area and tends to fill it with the HU values of the tissue, CycleGAN and CUT tend to generate additional air pockets in the case of extreme air volumes in a patient, as shown in the axial and sagittal slices. The window width and window level of the shown CT and sCT images are 844 and ‒100 HU.

The image similarity metrics (Table 1, see RQ2 columns) revealed that there were no drastic differences in MAE mean ± SD, which were 67.58 ± 18.99 HU for Pix2Pix, 66.97 ± 18.81 HU for CycleGAN, and 69.49 ± 19.40 HU for CUT. However, CUT performed slightly better in the bone areas, with a bone MAE mean of 259.93 ± 50.25 HU. Figure 4 presents the analysis of DVH differences, with mean differences below 1% for all models, while outliers above 2% for CycleGAN and CUT. Lower (*p <* 0.05) DVH differences were observed for the Pix2Pix, trained in paired fashion, particularly in the mean values of target and OAR objectives. For example, for Stomach D2%, the mean differences were ‒0.03%, ‒0.73%, and ‒0.70% for Pix2Pix, CycleGAN, and CUT, respectively. Figure 5 further supports these dosimetric findings, showing a case with extreme air pockets.

### 3.3 Effect of NN input‐output channels configuration (RQ3)

**FIGURE 6:**
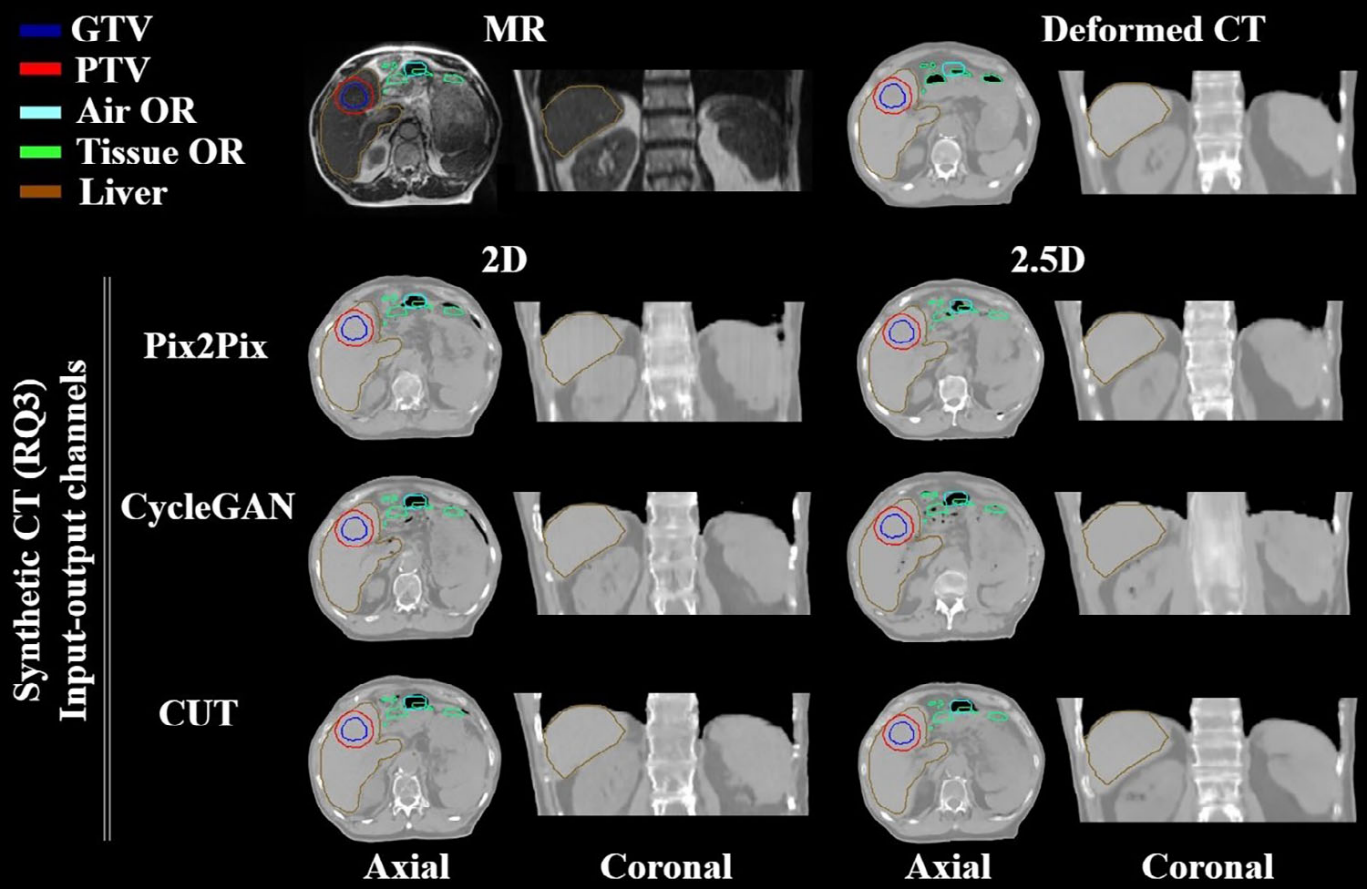
Research question 3. Examples of sCT volumes generated utilizing 2D and 2.5D input‐output channel configurations for a liver case. Air OR and Tissue OR contours define regions for air and soft tissue density overrides (OR), ensuring correct placement on deformed CT relative to MR for treatment planning. The Pix2Pix 2.5D configuration induced a strong improvement in the area of the spine. However, for architectures trained in an unpaired fashion, additional distortions were observed for 2.5D: intense blurriness for CycleGAN and for CUT caused liver enlargement. The window width and window level of the shown CT and sCT images are 844 and ‐100 HU.

The third experiment, which analyzed NN input‐output channel configurations, showed that the 2.5D configuration improved Pix2Pix performance, reducing discontinuities in the bone region (Figure 6). The best results across all NN models (Table 1, see RQ3 columns) were achieved with the 2.5D Pix2Pix model, yielding a mean MAE ± SD of 63.21 ± 18.47 HU compared to 67.58 ± 18.99 HU for 2D. Bone MAE was lower as well. Moreover, no DVH outliers were observed for 2.5D Pix2Pix (Figure S6), although differences were not statistically significant. For architectures trained in unpaired fashion, 2.5D configuration yielded poorer results, with more outliers in target DVH for CUT. CycleGAN results showed severe blurring and distortion of bone and body contours (Figure 6).

### 3.4 Training set size (RQ4)

**FIGURE 7:**
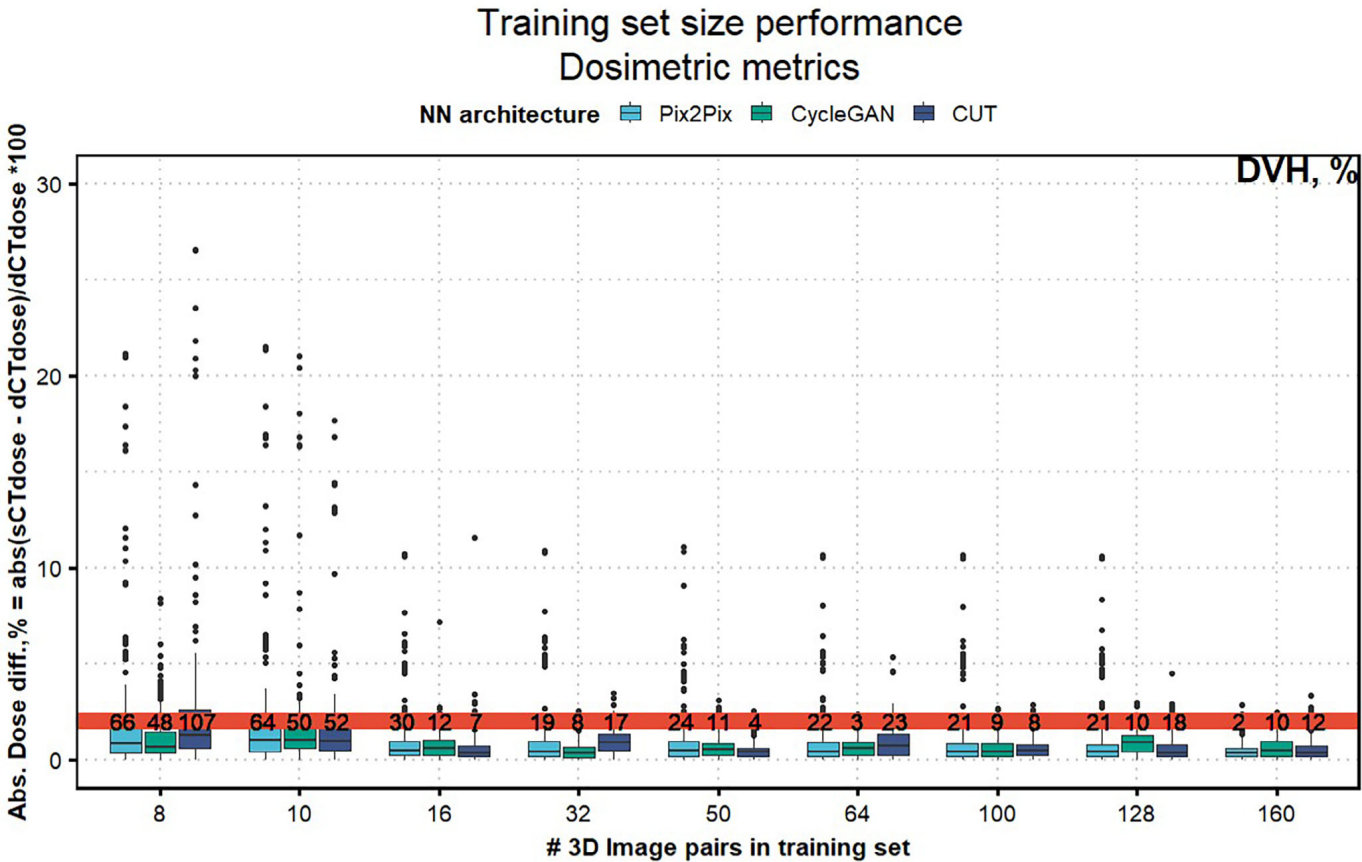
Research question 4. The analysis shows how dosimetric metrics, including all DVH parameters, evolve as the size of the training set increases (40 axial slices per 3D volume). The test cohorts remained the same for all models. The number of outliers above 2% is shown on the red line for each training set size.

The evolution of DVH differences and image similarity metrics with an increase in training size is shown in Figure 7 and Figure S8, respectively. The results of the shuffle‐split cross‐validation demonstrated the stability of the network training (Figure S7).

### 3.5 Correlation of image similarity with dosimetric metrics (RQ5)

**FIGURE 8:**
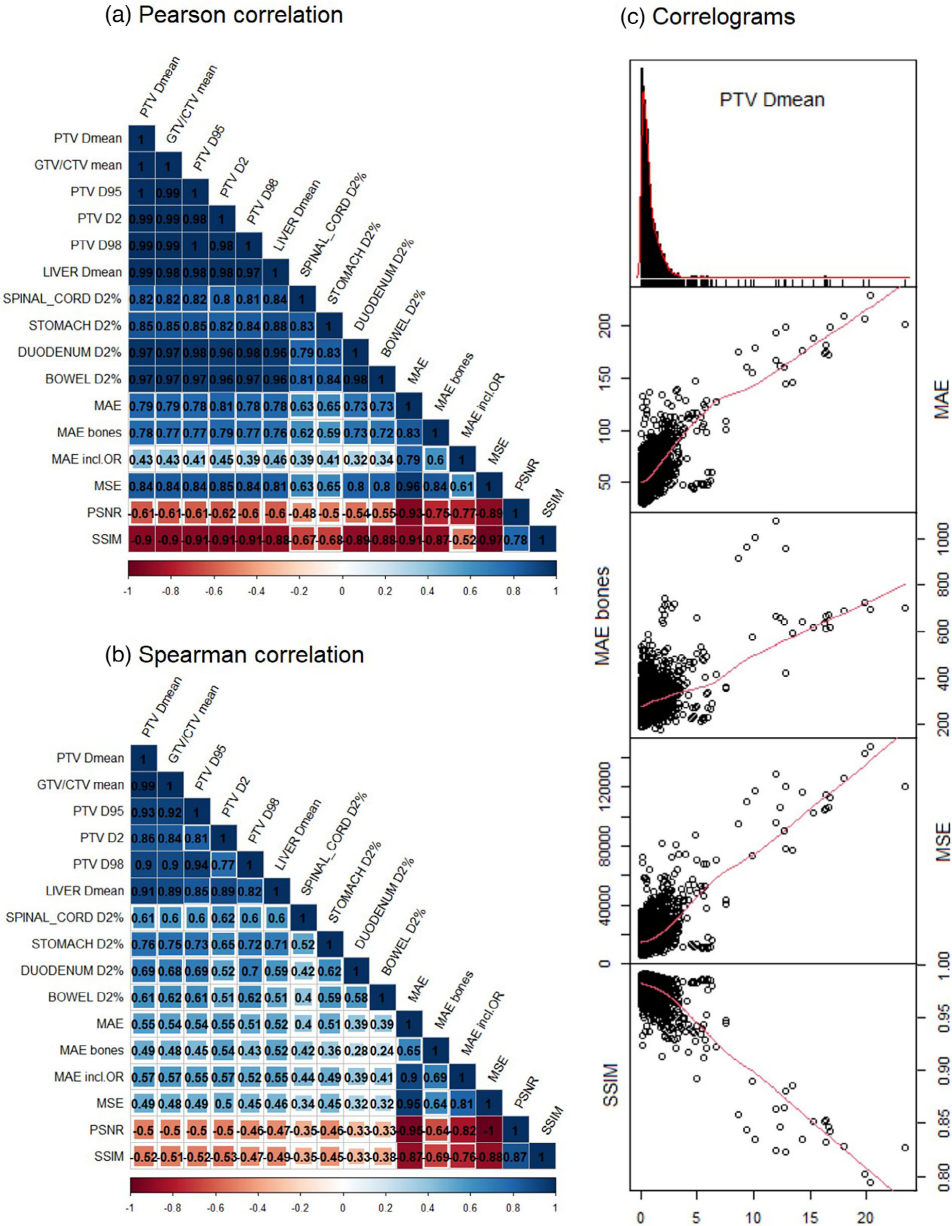
Research question 5. Pearson (a) and Spearman (b) rank correlation coefficient between image similarity and dosimetric metrics (stronger correlation—darker color) are shown on the left side. Correlation diagrams (c) between PTV Dmean difference (Abs. Dose diff., % = abs(sCTdose—dCTdose)/dCTdose *100) and strongly correlated image similarity metrics (MAE, MAE Bones, MSE, SSIM) are shown on the right. The red line on the scatterplots is a Locally Estimated Scatterplot Smoothing (LOESS) curve, which visually represents how the values of one variable are related to the values of another variable in a local, non‐parametric manner. Straight red line indicates a more consistent linear relationship between the metrics.

Figure 8 shows the Pearson and Spearman correlation coefficients for image similarity with dosimetric DVH metrics and correlograms. The correlograms revealed the presence of noise in linear relationships, particularly where the absolute deviations in DVH parameters were less than 5%. In the area of larger deviations, a more linear relation can be observed. Estimation of metrics from NN models trained on diverse ranges of patient image volumes (8 to 160) showed strong correlation of MAE, MAE bones, MSE, SSIM with the target DVH parameters based on Pearson correlation coefficients, with absolute values ranging from 0.77 to 0.91 for different target DVH parameters. At the same time, Spearman correlation coefficient, which analyzes non‐linear dependencies, revealed moderate correlations between these metrics with absolute values between 0.43 and 0.57. All correlation coefficients showed to be statistically significant^44^ at the 0.05 significance level. At the same time, no strong correlation was found when focusing solely on the metrics of the best‐performing networks trained with 160 patients (Figure S9).

## 4 DISCUSSION

This study aimed at providing a comprehensive evaluation of GAN‐based approaches for the generation of sCT in the abdominal region, an area that has been relatively underexplored compared to other anatomical regions, considering various key factors that influence the quality and clinical applicability of sCTs. Previous research in this area has largely focused on evaluation of isolated parameters^28^, ^50^ or relied heavily on image similarity metrics.^23^, ^29^, ^30^ Many researchers emphasized the issue of interpretability of image similarity metrics and the use of these metrics as benchmarks between different studies, requiring complex assessment of different parameters within the same study^21^ or organization of specific hackathon challenges.^32^ To address these limitations, we established an automated pipeline that integrates both image similarity and dosimetric metrics, enabling a more robust assessment of sCT quality and bridging the gap between technical performance and clinical relevance.

The results of the first experiment evaluating the most widely used generator architecture in medical image analysis, U‐Net, and ResNet architecture suggest that ResNet enhances sCT generation, particularly in challenging regions like bones (15% MAE improvement) and organ boundaries. We attribute this effect to the differences in the ways generators transform the images: U‐Net's skip connections^38^ blend the information of high‐level and low‐level features, which can lead to the loss of fine details. In contrast, ResNet could avoid these problems^51^, ^52^ as it passes information within the same transformation level (Figure S2). Additionally, ResNet has shown to be helpful in reducing outliers in dose distribution estimation. While it is important to evaluate the performance of the networks by finding the models that are able to minimize the mean dose differences between dCT and sCT, we consider the sensitivity of the DL methods and absence of outliers in DVH differences for under‐represented cases^53^ to be among the most important parameters in clinical practice. Future research directions could include the direct incorporation of dosimetric metrics into the loss functions and irradiation maps in the area of evaluation of image similarity metrics. A limitation of this study is that ResNet was only tested with Pix2Pix, requiring further evaluation with other GANs.

The second experiment, comparing paired (Pix2Pix) and unpaired (CycleGAN, CUT) architectures, showed that Pix2Pix outperforms the other architectures in terms of dosimetric accuracy, but suffered from structural smoothing. In contrast, CycleGAN and CUT, trained in an unpaired fashion, preserved better anatomical details, especially for bones and air inclusions. We attribute this to the lack of ground truth with perfect contour alignment between MR and dCT, especially in a region as difficult to co‐register as the abdomen, which unpaired GANs, focused on higher‐level commonalities, can handle better.

Concerning the number of input‐output channels, a strong performance improvement, based on image similarity metrics, is found for the 2.5D approach for the Pix2Pix architecture (ResNet generator). This shows the best performance among all models in the study. Other studies have shown similar results.^29^, ^35^ It might be attributed to the increase in the network perceptive field for each output pixel in a pixel‐to‐pixel translation network. While the results for the unpaired NN architectures showed increased blurring under the 2.5D configuration—especially for CycleGAN—performance degradation may be attributed to a larger number of learnable parameters, insufficient training set size, and potential failures in generating consistent outputs for consecutive frames.

The fourth experiment revealed that neural networks may generate sCT with mean DVH differences within the clinically acceptable range of 2%, even with a very small training set. This result is in line with many analyses done on small patient cohorts.^21^, ^30^, ^35^ However, in small test sets potential outliers may go unnoticed, leading to an overly optimistic assessment of model performance. Increasing the training set size helps to drastically reduce the occurrence of dosimetric outliers, with 160 image volumes resulting in the fewest outliers, and improves model generalizability, especially for unseen anatomies or imaging artifacts.

Our findings demonstrate that image similarity metrics are ineffective for predicting small DVH deviations within the clinically relevant range of ± 2%. We attribute this noise to the absence of absolute ground truth in sCT generation. Since CT images are acquired after MR images, and considering the challenges of organ and air bubble motion, deformable registration can introduce errors.^10^ As a result, image similarity metrics reflect not only network performance but also uncertainties in registration, leading to dosimetric deviations from zero, especially in high‐performance scenarios. Previous studies^32^ and our analysis of networks trained with 160 patients (Figure S9) have similarly shown no strong correlation for the best‐performing models. This emphasizes that dosimetric metrics remain the gold standard, and identifying robust, resource‐efficient alternatives remains an open challenge.

In our fifth experiment, we expanded the correlation assessment by including sCT metrics from NN models trained on diverse ranges of image volumes (8 to 160). This approach revealed that there is correlation of image similarity metrics with DVH metrics in the area of poor image quality—a relationship not previously reported in the literature. Specifically, metrics such as MAE, MAE bones, SSIM could be effectively used to determine large deviations in DVH above 5% (Figure 8). Although MSE showed strong correlations in low‐quality scenarios, its sensitivity to outliers and non‐linear response to change in the input‐output channel configuration (2.5D) make it less suitable for primary screening compared to MAE. Notably, MAE in the bone area showed one of the strongest correlations, despite its limited use in previous research. This is likely due to the challenges of visualizing bones, particularly the ribs, in abdominal MRI. If these challenging regions are well‐reconstructed, the overall quality is likely high. Our findings suggest that the aforementioned image similarity metrics could serve as practical surrogates for dosimetric metrics during NN model development, offering a preliminary screening tool to flag sCTs with substantial reconstruction errors, and promoting more consistent reporting across studies.

A major limitation of this study is the dataset formation, consisting exclusively of abdominal images. In contrast to most studies,^26^, ^27^, ^35^ no manual exclusion of 3D volumes was performed, except for patients having metal implants. It is a single center, single scanner study. This may limit the generalizability of the model, especially when applied to more heterogeneous data sets. Additional data augmentation could be performed to account for sample variability. Finally, the dosimetric assessment in this study is specific to photon treatments and should not be extended to proton treatments, which are known to be more sensitive to dCT‐sCT differences along the beam path.

Future work will focus on developing robust models capable of generating reliable sCT images independent of the treatment site, taking advantage of the similar tissue intensities in all body formations, and application to multicenter studies. In addition, further research is needed on the interpretability aspect of DL networks for sCT generation, as well as refinement of the loss function and performance parameter estimation of networks focused solely on distribution areas of irradiation in addition to the body masking approach.^54^ Furthermore, the synergistic effect found between the independent architectures needs to be investigated, including evaluation of recently emerged diffusion models,^55^ vision transformers^24^ and application of ensemble learning^56^ for sCT generation task. Finally, finding ways to implement an automated quality assurance procedure is fundamental for the clinical applicability of the method.

## 5 CONCLUSION

The present study provided a comprehensive comparison of GANs for sCT generation in the sparsely studied abdominal area and evaluated the impact of its key parameters, namely GAN architectures, specific generator architectures, number of input‐output channels and training set size. Trained in paired fashion Pix2Pix model: using 2.5D input‐output channels, a ResNet generator, and no MR image normalization; emerged as the best‐performing model, further advancing the state‐of‐the‐art with a mean MAE of 63.21 HU, a GTV/CTV Dmean difference of ‐0.15% from the original plan, and no outliers above 2%. The proposed DL‐based sCT generation methods are clinically applicable for treatment planning in the abdominal area, with mean DVH indicator discrepancies from the original dCT‐based plan of less than 0.8% for the best‐performing models, including unpaired trained in 2D CycleGAN and CUT. Increasing the training size has proven to be essential to avoid outliers in dosimetric calculations. While our results confirmed that image similarity metrics can not reliably predict small DVH deviations within a clinical threshold of ± 2%, this study contributes identifying specific metrics (MAE, MAE bones, and SSIM) that correlate with DVH parameters and can detect significant deviations above 5%. These metrics offer valuable support for the training and evaluation, while having the potential of improving consistency of reporting across studies.

## CONFLICT OF INTEREST STATEMENT

The Department of Radiation Oncology, University Hospital Zurich has teaching and research agreements with Siemens Healthineers. University Hospital Zurich had teaching and research agreements with Viewray Inc.

## Supporting information

Supporting Information
