# Supplementary material for: A comprehensive comparative study of generative adversarial network architectures for synthetic computed tomography generation in the abdomen
Source: Med Phys. 2025 Aug 13;52(8):e18038. doi: 10.1002/mp.18038 (PMC12351101; doi:10.1002/mp.18038)
Supplement: Supplementary file 1 — Supporting Information [file MP-52-0-s001.docx]

# Supplementary Material

Supplementary Table S1: Distribution of patients by treatment sites in the training and test sets

| Treatment site | Number of image pairs  (number of patients) | | |
| --- | --- | --- | --- |
|  | Training | Test | Grand Total |
| Abdomen | 43 (35) | 10 (10) | 53(45) |
| Adrenal gland | 25 (21) | 5 (5) | 30 (26) |
| Kidney | 10(8) | 3(3) | 13(11) |
| Liver | 62(49) | 14(14) | 76(63) |
| Pancreas | 18(18) | 4(4) | 22(22) |
| Spleen | 2(2) | 1(1) | 3(3) |
| Grand Total | 160(117) | 37(37) | 197(154) |


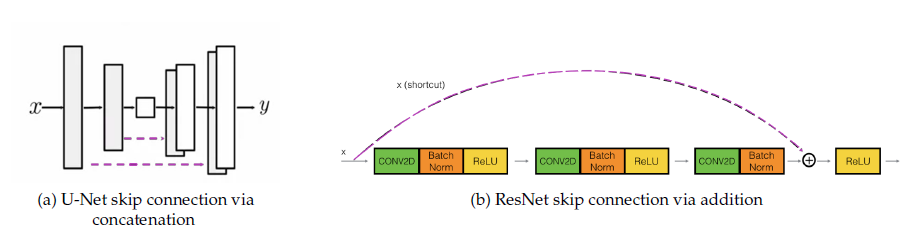


Supplementary Figure S2: Skip connections in different GAN generators. The violet arrows show the differences in skip connections between the U-Net architecture (a) and the ResNet (b). The U-Net^1^ generator employs symmetrically designed encoder and decoder pathways, where outputs of the encoder layers are directly added to the inputs of the decoder layers (skip connection) to recover spatial information lost during downsampling. In contrast, the skip connection in ResNet^2^ the skip connection addresses the vanishing gradient problem by providing an alternative path for gradient to flow through, which is why it skips any two consecutive convolutions within the downsampling or upsampling operations themselves thus “flattening” the model. U-Net for 256x256 input images and ResNet with 9 building blocks were utilized in the experiments^3,4^.


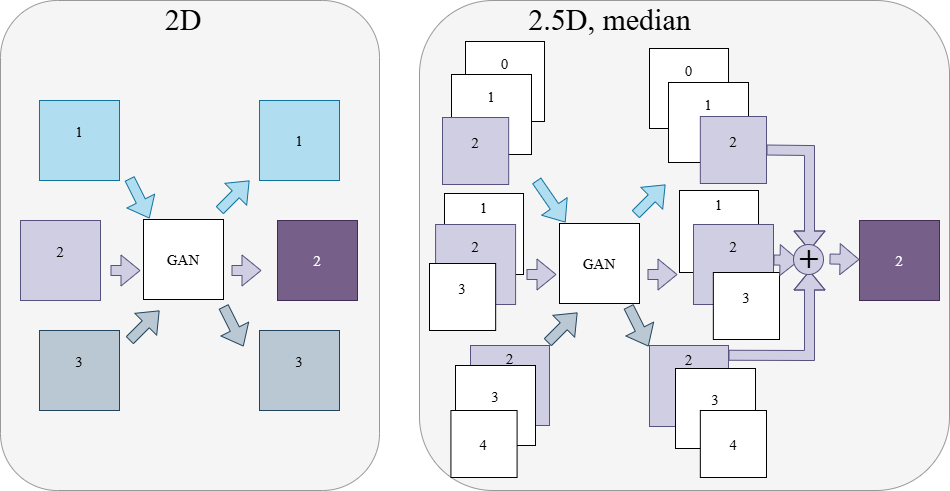

Supplementary Figure S3: Schematic representation of the network configuration: number of input/output channels, using the end-to-end journey of Slice 2 (dark purple rectangle) of the resulting 3D volume as an example. When testing the network, in the 2D approach, the real MR slice 2 image is passed to the network as a single-channel input, and the sCT image of slice 2 is generated as a single-channel output, which is further directly passed for the creation of a patient’s 3D DICOM volume and the evaluation of the quality of the network training. In the 2.5D approach employed in this study, 3 sequential axial MR slices with stride 1 in axial dimension are passed as three-channel input, and the sCT images of 3 sequential axial slices are generated as three-channel output. Then, for combining the results in the median approach, sCT slice 2 (dark purple rectangle) is composed out of all NN passes, where all occurrences of MR slice 2 in the input are taken from the matched output (all light purple output rectangles) and combined using a 1*1*3 median filter with the same weight for each slice position in the output.

Supplementary Table S4: Hyperparameters of NN models, used in all experiments. For optimizing the hyperparameters, a validation set has been used, which consists of 20% of the training set sampled with the same conditions as the test set.

| Hyperparameter | NN model | | |
| --- | --- | --- | --- |
|  | Pix2Pix | CycleGAN | CUT |
| GAN mode | vanilla  (the cross-entropy objective used in the original GAN paper, L1 loss,  lambda_L1 300) | vanilla (the cross-entropy objective used in the original GAN paper, L1 loss, lambda_A 20, lambda_B 20, Lambda_Identity 0) | non-saturating (a subtle variation of the standard loss function is used where the generator maximizes the log of the discriminator probabilities, L1 loss, Lambda_NCE 1, Lambda_GAN 2) |
| Batch size | 1 | 1 | 1 |
| Size of image buffer^5^ | 20 | 80 | 40 |
| Optimizer | Adam | Adam | Adam |
| Learning rate | 0.0002 | 0.0002 | 0.0002 |

Supplementary Table S5: Experimental design and selection of appropriate statistical methods. All experiments employ a repeated measures design, where sCTs are generated using different NN architectures or training approaches for the same cohort of patients. The resulting data from these experiments is non-normally distributed and contains outliers, necessitating the use of non-parametric statistical tests.

| RQ | Hypothesis | Independent within-subject variable | Dependent variable | Appropriate statistical test |
| --- | --- | --- | --- | --- |
| RQ1 | H0: The choice of NN generator has no effect on sCT generation, as measured by deviation of DVH parameters.  H0​: μ(G:U-Net)​$=$μ(G:ResNet)​  H1: ​μ(G:U-Net)​$\neq$μ(G:ResNet)​ | Generator of a NN (U-Net, ResNet): single factor with **two** levels | Irradiation dose deviation, DVH parameters, each evaluated independently | Wilcoxon-Pratt test |
| RQ2 | H0: The choice of NN architecture has no effect on sCT generation, as measured by deviation of DVH parameters.  H0​:μ(Pix2Pix)​$=$μ(CycleGAN)$=$μ(CUT)​  H1: at least one ​μ differ | NN architecture (Pix2Pix, CycleGAN, CUT): single factor with **three** levels | Irradiation dose deviation, DVH parameters, each evaluated independently | Friedman test |
| RQ3 | H0: The choice of NN input-output channels configuration of a NN model has no effect on sCT generation, as measured by deviation of DVH parameters.  H0​: μ(2D)​$=$μ(2.5D)  H1: at least one ​μ differ | NN input-output channels configuration (2D, 2.5D): single factor with **two** levels | Irradiation dose deviation consists of two vectors of multiple DVH parameters that represent the accumulated dose values for the target (tumor PTV/GTV) and AOR (e.g. liver). | PERMANOVA |


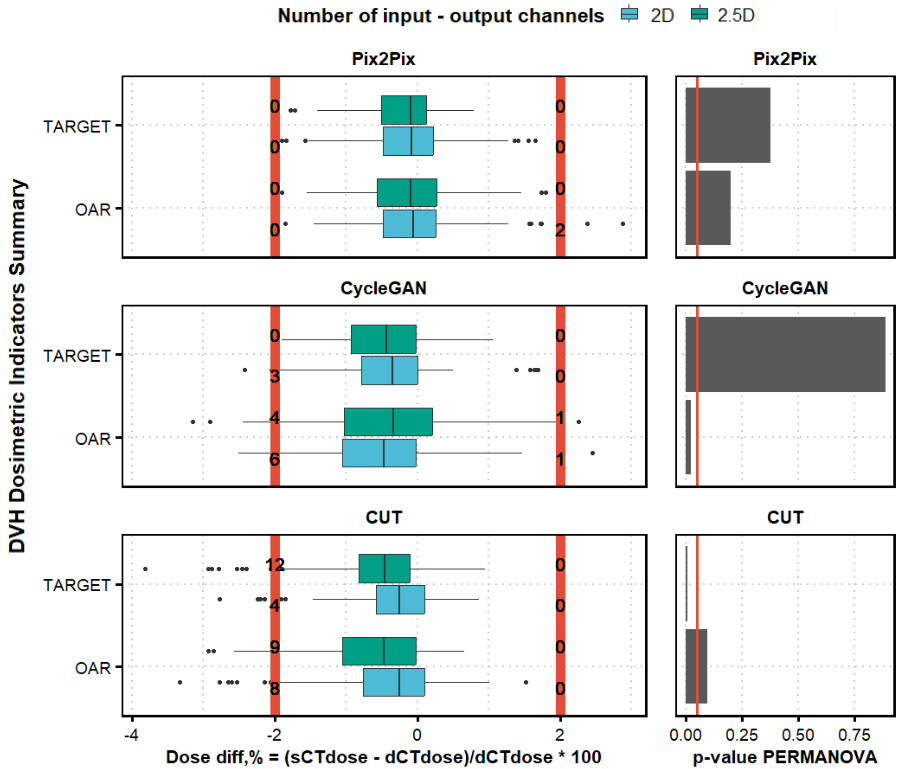

Supplementary Figure S6: Research question 3. Differences in DVH dosimetric indicators between plans calculated on dCT and sCT, generated with Pix2Pix, CycleGAN and CUT models, employing different numbers of input-output channels: 2D - in blue, 2.5D - in green. The number of outliers below -2% and above 2% is shown next to the red lines for each group of DVH indicators. The right panel reports the results of the PERMANOVA test (Supplementary Table S5). The significance level p=0.05 is highlighted with a vertical line. Most outliers were found in the bowel and stomach calculations for OAR-DVH differences, likely due to the inclusion of cases with numerous air pockets.


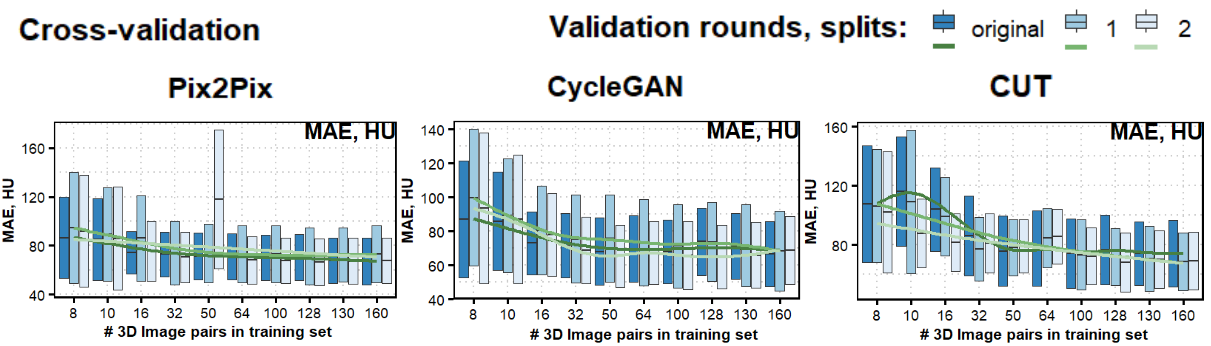
Supplementary Figure S7: Results of shuffle-split cross-validation demonstrating the stability of network performance, using the example of evaluating the effect of training set size for Pix2Pix, CycleGAN and CUT. Three splits were used: original, results reported in the paper (dark blue bars, dark green line connecting means of image similarity metrics), 1st additional split (blue bars, green line), and 2nd additional split (light blue bars, light green line). No significant differences were found, demonstrating the validity of the results. The outlying values in the 2nd validation round with 50 3D image pairs for Pix2Pix can be attributed to server errors during the training process.


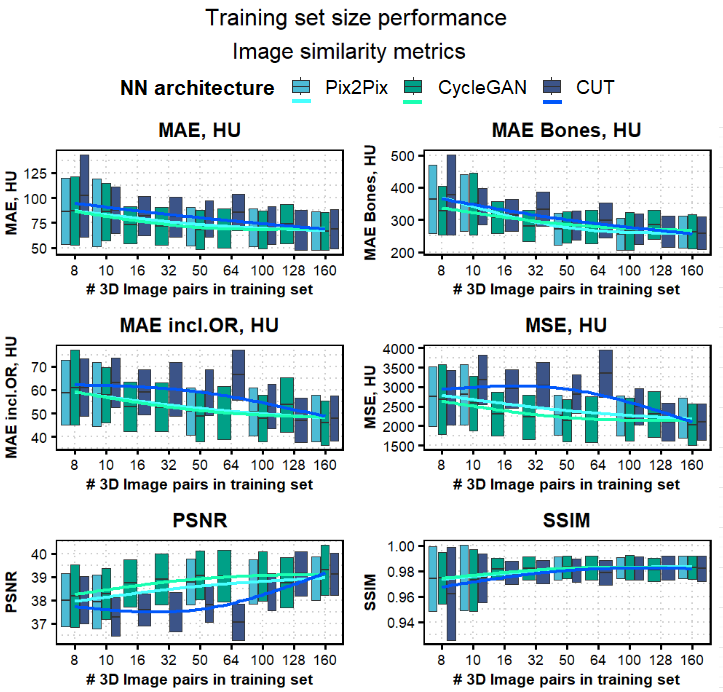


Supplementary Figure S8: Research question 4. The analysis shows how image similarity metrics evolve as the size of the training set increases (40 axial slices per 3D image volume). The test cohorts remained the same for all models. Performance improvements with increasing training data are better reflected in the image similarity metrics, with the MAE decreasing from a mean of 86.45 HU to 67.58 HU with PixPix (trained in paired fashion), from 86.98 HU to 66.97 HU with CycleGAN and from 102.07 HU to 69.49 HU with CUT for the models trained on 8 and 160 3d image volumes (trained in unpaired fashion), respectively, while simultaneously reducing the number of outliers in the dosimetric metrics. While the Pix2Pix and CycleGAN image similarity related training curves reached their plateau at 100-160 3D image volumes in the training set, the CUT architecture continued its performance improvement and maintained a steeper training set size performance curve.


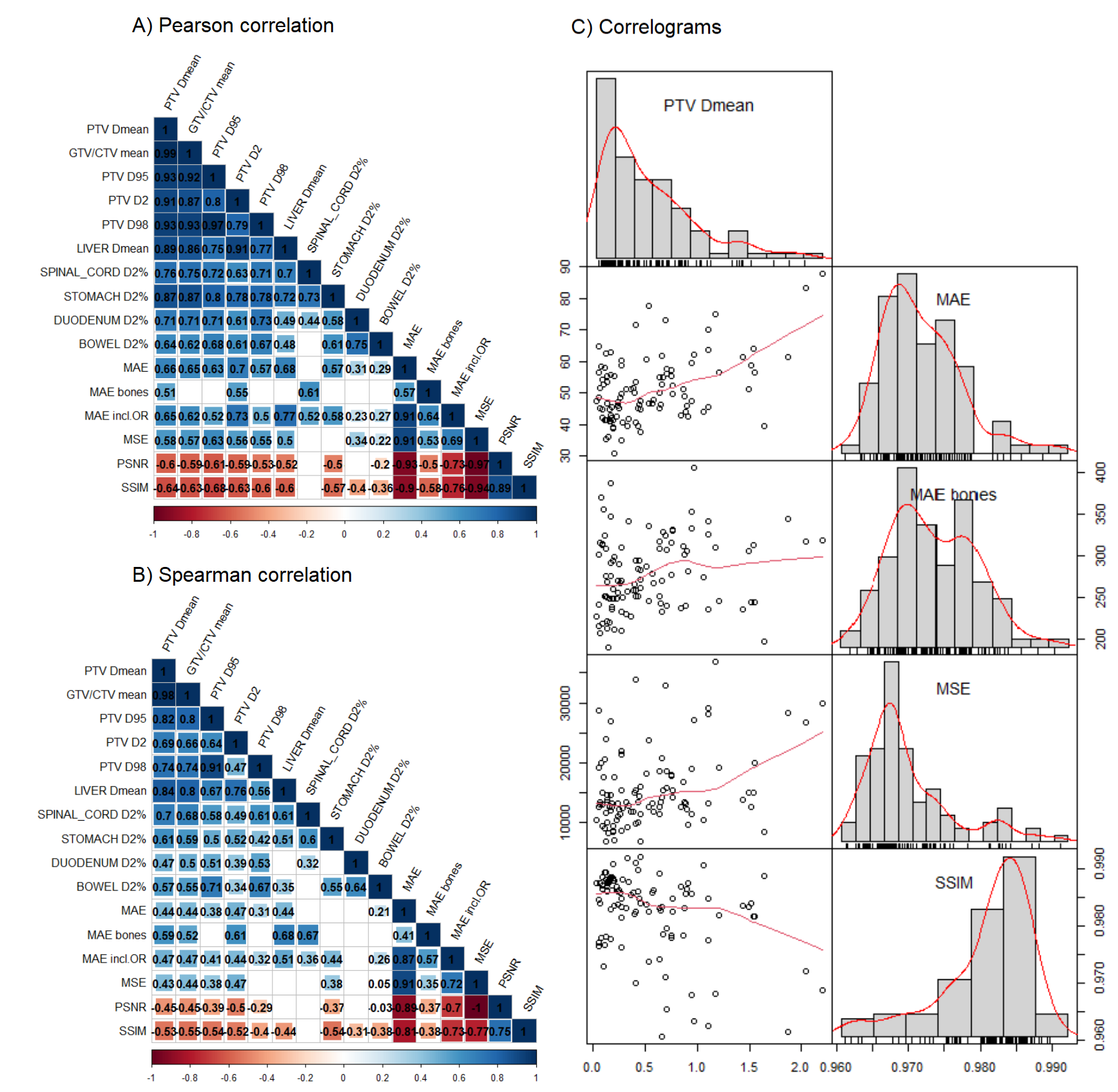


Supplementary Figure S9: Pearson (A) and Spearman (B) rank correlation coefficient between image similarity and dosimetric metrics (stronger correlation - darker color, empty squares for non-significant correlation p>0.05) for NN models *with 160 patients in the training set* are shown on the left side. Correlation diagrams (C) between PTV Dmean difference (Abs. Dose diff., % = abs(sCTdose - dCTdose)/dCTdose *100) and image similarity metrics (MAE, MAE Bones, MSE, SSIM) are shown on the right. The red line on the scatterplots is a Locally Estimated Scatterplot Smoothing (LOESS) curve, which visually represents how the values of one variable are related to the values of another variable in a local, non-parametric manner. No strong correlation was found between image similarity metrics and target DVH indicators, while evaluating only best performing models.

# References

1. Ronneberger O, Fischer P, Brox T. U-Net: Convolutional Networks for Biomedical Image Segmentation. In: Navab N, Hornegger J, Wells WM, Frangi AF, eds. *Medical Image Computing and Computer-Assisted Intervention – MICCAI 2015*. Springer International Publishing; 2015:234-241. doi:10.1007/978-3-319-24574-4_28

2. He K, Zhang X, Ren S, Sun J. Deep Residual Learning for Image Recognition. In: *2016 IEEE Conference on Computer Vision and Pattern Recognition (CVPR)*. ; 2016:770-778. doi:10.1109/CVPR.2016.90

3. Zhu JY. junyanz/pytorch-CycleGAN-and-pix2pix. Published online October 1, 2024. Accessed October 1, 2024. https://github.com/junyanz/pytorch-CycleGAN-and-pix2pix

4. Lapaeva M. MR-based synthetic CT generation for MR-guided radiotherapy. *Master’s Thesis*. https://www.merlin.uzh.ch/contributionDocument/download/15045

5. Shrivastava A, Pfister T, Tuzel O, Susskind J, Wang W, Webb R. Learning from Simulated and Unsupervised Images through Adversarial Training. In: *2017 IEEE Conference on Computer Vision and Pattern Recognition (CVPR)*. ; 2017:2242-2251. doi:10.1109/CVPR.2017.241
